# Construction of ultrasound-responsive urokinase precise controlled-release nanoliposome applied for thrombolysis

**DOI:** 10.3389/fbioe.2022.923365

**Published:** 2022-08-09

**Authors:** Yongliang Fan, Li Liu, Fang Li, Hang Zhou, Yizhou Ye, Chunping Yuan, Hongli Shan, Wangfu Zang, Yu Luo, Sijing Yan

**Affiliations:** ^1^ Department of Cardio-Thoracic Surgery, Shanghai 10th People’s Hospital, School of Clinical Medicine of Nanjing Medical University, Shanghai, China; ^2^ Department of Cardiovascular Surgery, Shanghai General Hospital, Shanghai Jiao Tong University School of Medicine, Shanghai, China; ^3^ Department of Ultrasound Medicine, Chongqing University Cancer Hospital, Chongqing, China; ^4^ Shanghai Engineering Technology Research Center for Pharmaceutical Intelligent Equipment, Shanghai Frontiers Science Center for Druggability of Cardiovascular Non-coding RNA, Institute for Frontier Medical Technology, Shanghai University of Engineering Science, Shanghai, China; ^5^ Department of Ultrasound, Chongqing Hospital of Traditional Chinese Medicine, Chongqing, China

**Keywords:** pulmonary embolism, urokinase, sonodynamic, protoporphyrin (PPIX), singlet oxygen

## Abstract

Urokinase is widely used in the dissolution of an acute pulmonary embolism due to its high biocatalytic effect. However, how to precisely regulate its dose, avoid the side effects of hemolysis or ineffective thrombolysis caused by too high or too low a dose, and seize the golden time of acute pulmonary embolism are the key factors for its clinical promotion. Therefore, based on the precise design of a molecular structure, an ultrasonic-responsive nanoliposome capsule was prepared in this paper. Singlet oxygen is continuously generated under the interaction of the ultrasonic cavitation effect and the sonosensitizer protoporphyrin, and the generated singlet oxygen will break the thiol acetone bond between the hydrophilic head and the hydrophobic tail of the liposome, and the lipid The body structure disintegrates rapidly, and the urokinase encapsulated inside is rapidly released, down-regulating the expression of fibrinogen in the body, and exerting a thrombolytic function. The *in vitro* and *in vivo* results show that the smart urokinase nanoliposomes prepared by us have sensitive and responsive cytocompatibility to ultrasound and good *in vivo* thrombolytic properties for acute pulmonary embolism, which provides a new strategy for clinical acute pulmonary embolism thrombolysis.

## 1 Introduction

Pulmonary embolism (PE) refers to diseases or clinical syndromes caused by various emboli blocking the pulmonary artery or its branches, including pulmonary thromboembolism (PTE), fat embolism, amniotic fluid embolism, tumor embolism, etc (Konstantinides et al., 2019; van der Pol et al., 2019; Barco et al., 2020). Pulmonary thromboembolism is the most common type, commonly referred to as acute pulmonary embolism (APE) (Shonyela et al., 2015; Opris et al., 2017). Pulmonary embolism is often secondary to deep venous thrombosis (DVT), which is essentially a clinical manifestation of the same disease at different stages, collectively referred to as venous thromboembolism (VTE) (Stein et al., 2010; Kaditis and Alexopoulos, 2021).

The fatality rate of APE is also high, ranking third in Western countries after myocardial infarction and malignant tumors. It is estimated that about 10% of APE die within 1 h after onset, and the fatality rate of APE without diagnosis and treatment can reach 30% (Kostrubiec et al., 2012). With early intervention and treatment, the fatality rate can be reduced to 2–8%. Therefore, APE is a disease with a high morbidity rate, high misdiagnosis rate, and high mortality rate, which needs to be paid attention to by clinicians.

The morbidity and mortality of PE patients under the age of 40 are much lower than those of the elderly, and insufficient attention has been paid to it (Abe et al., 2019). However, some studies have shown that APE has been proved to be an important cause of death in young people. A study by Sakuma *et al.* in 2007 examined Autopsy records and found that APE contributed more to deaths in patients aged 20–39 than in other age groups, accounting for 2.3% of deaths (Taniguchi et al., 2012). In 2008, an autopsy study of 1,000 patients with pulmonary embolism in India by Nandita *et al.* found that pulmonary embolism tends to be younger (Nandita and Vasistha, 2008). In 2010, Yamada *et al.* found that compared with Westerners, Japanese people with pulmonary embolism tend to be younger and more feminine (Yamada et al., 2010). Renda *et al.* calculated that the detection rate of APE in the U.S. adult population during CTPA-assisted examination increased from 0.621‰ to 1.123‰, the incidence rate increased by about 80%, and the mortality rate during the first 3 months after diagnosis was estimated up to 15% (Hayiroglu et al., 2015).

Although surgery can effectively remove thrombus in blood vessels, the operation is difficult and risky, and requires high medical equipment in the hospital and the medical skills of doctors; complex preoperative preparation is required; there are many postoperative complications; the cost is also high (Ronsivalle et al., 2013; Steglich-Arnholm et al., 2015; Izura Gómez et al., 2018). In addition to surgery, drug thrombolysis can also be used (Vedantham et al., 2017; Mazzolai et al., 2018; Naylor et al., 2018; Bolcal et al., 2019). So far, the clinically used thrombolytic drugs are mainly urokinase (UrokinaSe, United Kingdom). Goldhaber *et al.* have done a series of studies on thrombolysis in acute pulmonary embolism, confirmed the efficacy of United Kingdom and rt-PA, and compared the dose, time, route, and specific implementation methods of the drugs so that the treatment tends to be standardized (Goldhaber and Bounameaux, 2012; Huang et al., 2014). A large-scale study organized by Professor Wang Chen in China believes that 50 mg can not only receive a good curative effect but also reduce the risk of bleeding. Thrombolysis must consider the risk of bleeding, so patients with bleeding risk are contraindications to thrombolysis (Muñiz, 2012). Therefore, there is an urgent need to explore a new therapy with reliable efficacy, simple operation, low side effects, and complications for acute thromboembolism.

Sonodynamic therapy (SDT) developed in recent years is a new treatment modality based on ultrasonic excitation of sonosensitizers to trigger sonochemical reactions and generate highly toxic reactive oxygen species (ROS). Compared with photothermal/photodynamic therapy (Li et al., 2022), ultrasound has a deeper soft tissue penetration depth (≥10 cm) than light and has better potential for clinical application and translation (Chen et al., 2021; Hu et al., 2021; Xu et al., 2021; Xu and Pu, 2021). The application effect of SDT has been widely studied, such as the generation of reactive oxygen species by applying ultrasound to activate the sonosensitizer molecules hematoporphyrin, titanium dioxide (TiO_2_), *etc* (Gong et al., 2020). Clinical studies suggest that ultrasound can also accelerate thrombolysis, and is expected to be used for thrombus localization and blood flow monitoring.

Thereinto, this study intends to develop an advanced, safe and efficient thrombolysis technology by combining the advantages of deep tissue penetration of mechanical ultrasound and efficient thrombolysis of drug urokinase. Firstly, the nanocapsules are structurally modified. The ROS-sensitive (singlet oxygen) Linker is used to connect the hydrophilic and hydrophobic ends of the liposomes to obtain ultrasonic-responsive smart “nanocapsules”. The thrombolytic drug urokinase is enclosed inside the capsule. The prepared nanocapsules are injected into the body by intravenous injection, the embolism position is delineated by contrast CT imaging, and the ultrasound probe is aimed at the lesion site. The sonosensitizer in the main component of the nanocapsule liposome produces singlet oxygen and oxygen destruction under the action of ultrasound. The connection between the hydrophilic and hydrophobic liposomes and the collapse of the bilayer membrane structure. On the one hand, the precisely controlled release of the urokinase embolization site is achieved, the local drug concentration at the embolization site is increased, the embolization site is targeted, and the thrombolysis is rapid; on the other hand, it is loaded with The thrombolytic drug urokinase nanocapsules are not stimulated by exogenous ultrasound in other organs or tissues, the capsule structure remains intact, and urokinase is still “captured” in the capsule to avoid the risk of bleeding caused by systemic administration.

## 2 Experimental section

### 2.1 Materials and methods

Urokinase (United Kingdom) was obtained from Sigma-Aldrich Co. (Shanghai, China). DPPC, protoporphyrin (PpIX), DSPE. NH_2_, PEG_2k_-NH_2,_ and ^1^O_2_-cleavable linker modified DSPE-S(CH_3_)_2_-S-COOH were purchased from Shanghai Aicheng Biological Technology Co., Ltd. (Shanghai, China). Fetal bovine serum (FBS), Dulbecco’s Modified Eagle’s Medium (DMEM), RPMI 1640, penicillin, streptomycin, and 0.25% trypsin-EDTA were purchased from Gibco (New York, United States). Cell counting kit-8 (CCK-8) was purchased from Shanghai Aicheng Biological Technology Co., Ltd. (Shanghai, China).

### 2.2 Synthesis of ^1^O_2_-cleavable liposome fragment

***Synthesis of DSPE-PpIX:*** Briefly, PpIX (0.3 M) was dispersed in 10 ml of methanol and EDC (0.9 M, 1 ml methanol) was rapidly infused into the PpIX solution. Then, the mixed solution was stirred for 30 min. Secondly, NHS (0.9 M) dispersed in 1 ml methanol was rapidly added to the mentioned mixed solution, and mixed using magnetic stirring for 3 h. The active PpIX was added dropwise into DSPE-NH_2_ (0.1 M) dispersed in 10 ml of methanol at 300 rpm for 72 h. Finally, the product was then dialyzed against water using a dialysis bag (molecular weight cutoff of 5000 Da). The purified DSPE-PpIX was freeze-dried and stored at 4°C before use.

***Synthesis of DSPE-S-C*** (***CH***_***3***_)_***2***_ ***-S-PEG***_***2k***_ ***:*** The synthesis of DSPE-S-C(CH_3_)_2_-S-PEG_2k_ is similar to that of DSPE-PpIX, which is briefly described as follows: Briefly, DSPE-S-C(CH_3_)_2_-S-COOH (0.1 M) was dispersed in 20 ml of DMSO and EDC (0.3 M, 1 ml DMSO) was rapidly infused into the DSPE solution. Then, the mixed solution was stirred for 30 min. Secondly, NHS (0.3 M) dispersed in 1 ml DMSO was rapidly added to the mentioned mixed solution and mixed using magnetic stirring for 3 h. The active DSPE-S-C(CH_3_)_2_-S-COOH was added dropwise into PEG_2K_-NH_2_ (0.1 M) dispersed in 20 ml of DMSO at 300 rpm for 72 h. Finally, the product was then dialyzed against water using a dialysis bag (molecular weight cutoff of 8–14 KDa). The purified DSPE-S-C(CH_3_)_2_-S-PEG_2k_ was freeze-dried and stored at 4°C before use.

### 2.3 Synthesis and characterization of ultrasound-activated ULU nanoliposomes

DSPE–PpIX, DSPE-S-C(CH_3_)_2_-S-PEG_2k,_ and DPPC with a mass ratio of 5:25:1 were co-dissolved in 10 ml chloroform. The mixture solution was then evaporated to form a thin film using a rotary evaporator. Next, 20 ml of ultrapure water containing United Kingdom (2 mg) was added to the thin film and stirred at 55°C for 1 h. After the hydration process, the solution was sonicated in ice bath conditions for 60 min. The obtained solutions were then filtered using a 0.22-μm PVDF syringe-driven filter (Millipore, Bedford, United States) and then purified using ultrafiltration (cutoff molecular weight of 50,000 Da) at 5000 rpm three times to remove unloaded drugs. The obtained ULU nanoliposome (urokinase@lipsome, named as ULU) was stored at 4°C before use.

The ULU nanoliposome morphologies were imaged using JEM 2100F transmission electron microscope. The DLS and zeta potential of the obtained ULU nanoliposome were analyzed using the Zetasizer Nano series. UV-vis spectrophotometry was performed to investigate the Encapsulation Efficiency (EE, %) and Loading Efficiency (LE, %) of the United Kingdom (absorbance peak = 282 nm). The secondary structure changes before and after urokinase encapsulation were characterized by circular dichroism (CD). The colloidal stability of the ULU nanoliposome was evaluated using the Zetasizer Nano series. ULU nanoliposome dissolved in 1 × PBS was continuously observed for 7 days, and the corresponding DLS and PDI of the nanoparticles were recorded within 7 days.

### 2.4 Ultrasound activated release of United Kingdom

Singlet oxygen (^1^O_2_) was tested by ESR spectroscopy, see our previous work for details (Liang et al., 2020; Yang et al., 2021). ULU nanoliposome (25 mg) in 25 ml PBS buffer was irradiated for 0–5 min (1 W cm^−2^) and placed into a 50 ml flat-bottom centrifuge tube. After reaching the set ultrasonic time point, the solution was transferred to a high-speed centrifuge tube, centrifuged at 13,000 rpm for 10 min, the supernatant was taken to test the UV-Vis spectrum, and the cumulative release was calculated based on the UV-Vis absorption standard curve of urokinase. All assays were conducted three times.

### 2.5 Hemolysis and cytotoxicity evaluation of the ULU nanoliposomes

Hemolysis experiments were performed according to the previous work (Luo et al., 2013). Pig Pulmonary microvascular endothelial cells (PC-001) were used in the present study. PC-001 cells were cultured in complete DMEM, containing 10% FBS, 100 U mL^−1^ penicillin, and 0.1 mg ml^−1^ streptomycin. PC-001 cells were cultured at 37°C under humidified conditions with a 5% CO_2_ supply. PC-001 cells were plated in 96-well plates (5000 cells per well) and cultured overnight and then co-cultured with ULU at various concentrations (0, 10, 50, 100, 200, and 500 μg ml^−1^) for 24 h (0, 10, 50, 100, 200, and 500 μg ml^−1^ for 48 h). The cells were then washed three times with PBS and the CCK-8 agents were added to each well (10%). After 2 h coculture, the viabilities of PC-001 cells were measured at 450 nm using a microplate reader.

### 2.6 *In vivo* ultrasound performance of ULU nanoliposome

All animal experimental procedures were approved by the Ethical Committee of Shanghai 10th People’s Hospital. Rabbits with pulmonary embolism were randomly divided into five groups (*n* = 3). Pulmonary imaging was performed using Digital subtraction angiography (DSA) techniques and through a tail vein with indoxyl. The ULU nanoliposomes (urokinase dose of 2,000 units) were *in situ* injected into the pulmonary vein utilizing a jugular vein catheter. Then, the ultrasound was performed on the rabbits in the ultrasound-induced thrombolysis group (1 W/cm^2^, 5 min). After treatment, DSA imaging was used again to evaluate the efficiency of thrombolysis after pulmonary embolism. Blood samples were collected from the auricular vein to test the biochemical and blood routine indexes of rabbits to evaluate the changes in fibrin content before and after treatment.

### 2.7 *In vivo* biocompatibility assessment of ULU nanoliposome

All animal experimental procedures were approved by the Ethical Committee of Shanghai 10th People’s Hospital. The Kunming mice in each group were randomly divided into four groups. Major organs including heart, liver, spleen, lung, and kidney were collected and examined by H&E staining. The long-term biocompatibility of ULU nanoliposome was also assessed in healthy female Kunming mice. The mice in the treated groups were intravenously injected with 20 mg/kg of ULU nanoliposome. On day 0 and various post-injection time points (days 0, 30, 60, and 90), blood samples were examined for routine blood and biochemical analysis (*n* = 3). The corresponding major organs (heart, liver, spleen, lung, and kidney) were also examined by H&E staining at 0 and 90 days.

### 2.8 Statistical analysis

Data are shown as mean ± standard deviation. One-way analysis of variance (ANOVA) with Tukey’s multiple comparisons test was performed to analyze the statistical significance among different groups. Statistical significance was divided as three categories: *p < 0.05 **p < 0.01, and ***p < 0.001.

## 3 Results and discussion

### 3.1 Synthesis and characterization of ULU

**FIGURE 1:**
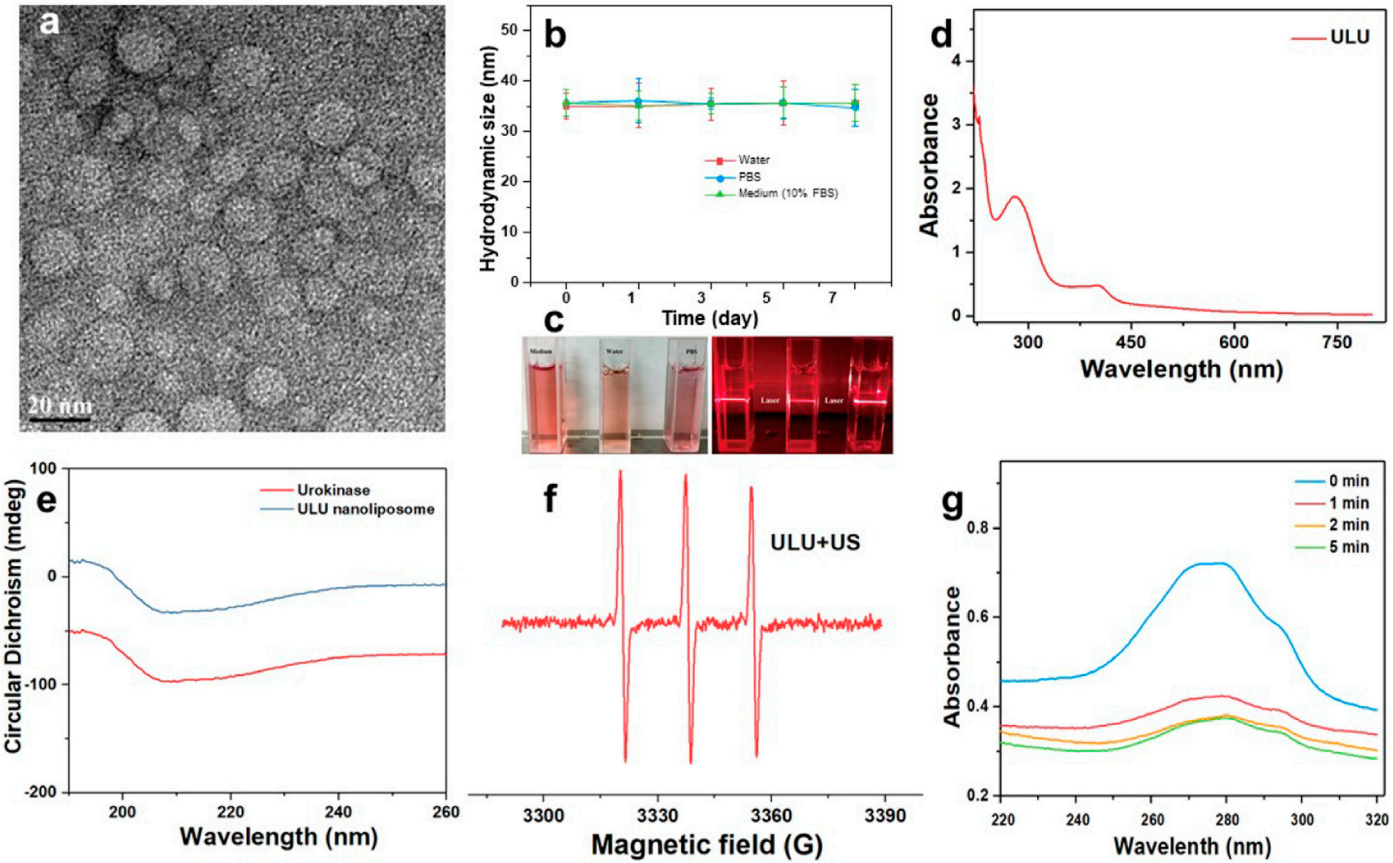
Transmission electron microscopy of ULU nanoliposome **(A)**, the hydrated particle size change **(B)**, and digital photographs **(C)** of colloidal stability of ULU nanoliposome dispersed in water, PBS, and cell culture medium (added 10% FBS) within 7 days; the UV-Vis absorption spectra of ULU nanoliposome **(D)**; Circular dichroism chromatogram of urokinase and the prepared ULU nanoliposome **(E)**; ESR spectra of ULU nanoliposome with the ultrasonic irradiation (1.0 W/cm^2^, 5 min, **(F)**; UV-Vis absorption spectra of urokinase-containing nanoliposome (ULU) at different time points after ultrasonic stimulation at 1.0 W/cm^2^, 5 min **(G)**.

The chemical structures of important fragments DSPE-S-C(CH_3_)_2_-S-PEG_2k_ and DSPE–PpIX of the synthetic ultrasound-responsive nanoliposomes were confirmed by ^1^H NMR spectroscopy. As shown in Supplementary Figure S1, the characteristic peaks attributed to singlet oxygen cleavage bond and polyethylene glycol -CH_2_CH_2_- exist in the prepared product of DSPE-S-C(CH_3_)_2_-S-PEG_2k_, indicating that the important fragments of ultrasonic-responsive nanoliposome were successfully synthesized. Sonodynamic-activated ULU was prepared using a film hydration method. In brief, a thin film composed of DSPE–PpIX, DSPE-S-C(CH_3_)_2_-S-PEG_2k_, and DPPC with a feeding mass ratio of 5:25:1 was synthesized and then hydrated with ultrapure water including the United Kingdom. The hydrophilic United Kingdom and hydrophilic were encapsulated into ROS-responsive liposomes through hydrophilic interactions. As shown in the transmission electron microscopy (TEM) images (Figure 1A), ULU was spherical vesicles and showed a uniform size distribution (diameter ≈30 nm). Dynamic light scattering (DLS) revealed that the hydrodynamic size of ULU was approximately 31.3 ± 1.4 nm (SI, Supplementary Table S1). Moreover, the polydispersity indexes (PDI) of ULU were 0.23, suggesting good mono-dispersity of the ULU nanoliposomes (Supplementary Table S1). The zeta potential of ULU was measured at -28.9 mV and showed an elevated surface charge, indicating successful United Kingdom loading. Furthermore, the prepared ULU nanoparticles showed excellent colloidal stability due to their stable hydrodynamic size and PDI over 7 days (Figures 1B,C). Given the protein properties of urokinase (United Kingdom), we used UV-vis spectrophotometry to qualitatively and quantitatively evaluate whether the nanoliposomes successfully encapsulated urokinase and the amount of encapsulation. As shown in Figure 1D, the characteristic UV absorption peak around 280 nm was attributed to the United Kingdom, indicating that urokinase was successfully encapsulated in nanoliposomes by the thin-film method and ultrasonic self-assembly. Combined with the standard curve of the United Kingdom, the Encapsulation Efficiency (EE) and Loading Efficiency of urokinase in ULU nanoliposome were calculated to be 91.8% and 5.9%, respectively (SI, Supplementary Table S2). The spectra of liposome nanocapsules before and after encapsulation of urokinase were analyzed by infrared spectroscopy (SI, Supplementary Figure S2). The results found that when the nanocapsule ULU is obtained by self-assembly of urokinase and liposome fragments by phacoemulsification method, the infrared spectrum is not only In addition to the characteristic absorption peaks of free urokinase, there are also absorption peaks of liposome fragments, indicating that we successfully encapsulated urokinase in liposome nanocapsules by phacoemulsification.

### 3.2 Ultrasound-induced drug release performance

Urokinase is essentially a protein, and changes in its secondary structure determine the biological activity of the enzyme. Therefore, we determined whether the biological activity of the enzyme was affected by the changes in the secondary results of urokinase before and after encapsulation by circular dichroism. The results showed that the secondary structure of urokinase did not change significantly before and after encapsulation, suggesting that its biological activity was maintained (Figure 1E).

The ultrasound-activated properties and ultrasound-induced release of United Kingdom from ULU were further investigated. Under conditions of ultrasound (1.0 W/cm^2^, 5 min), ULU nanoliposomes generate a large amount of singlet oxygen (Figure 1F), which further cuts the hydrophilic and hydrophobic ends of the improved nanoliposomes through singlet oxygen-sensitive linker. The nanoliposome capsule structure disintegrates and the urokinase encapsulated inside is released. UV-Vis absorption was performed on the supernatant before and after ultrasonic stimulation, combined with the UV absorption standard curve of urokinase, the cumulative release of the United Kingdom from ULU was 67.9% within 5 min (Figure 1G). The underlying mechanism can be explained as follows: Under ultrasonic excitation, protoporphyrins produce reactive oxygen species, which break the sensitive bonds in the liposome, and urokinase is released from the liposome nanospheres, resulting in the on-demand release of United Kingdom.

### 3.3 Hemolysis and cytotoxicity evaluation

**FIGURE 2:**
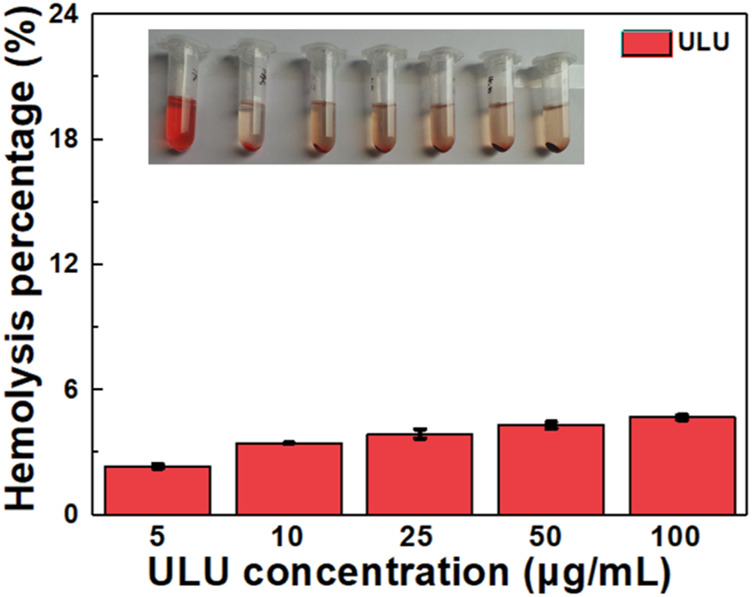
Hemolysis rate after 24 h co-culture of ULU nanoliposomes with erythrocytes.

**FIGURE 3:**
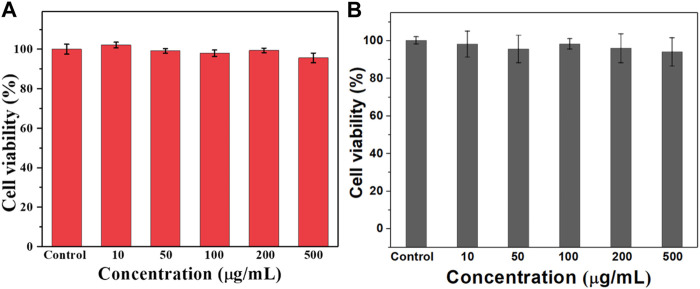
CCK8 was used to determine the survival rate of the nanoliposomes after co-culture with vascular endothelial cells for 24 h **(A)** and 48 h **(B)** to evaluate the cytotoxicity of the nanoliposomes.

Hemolysis is one of the important indicators to evaluate whether a drug can be administered intravenously. Therefore, we investigated the hemolysis by co-incubating the prepared ULU nanoliposomes with red blood cells. As shown in Figure 2, in the concentration range of 0–100 μg/ml, the hemolysis rate was lower than 5%. The results showed that ULU nanoliposomes have good blood compatibility, combined with *in vitro* ultrasonic stimulation to release urokinase. The results showed that, in the absence of external ultrasound, urokinase would not be released and would not cause hemolysis. Meanwhile, we co-cultured the prepared ULU nanoliposomes with vascular endothelial cells for 24 h and 48 h and tested the cell viability by the CCK8 method (Figures 3A,B). Within the concentration range (0–500 μg/ml), the cell viability remained above 90% even at up to 500 μg/ml, indicating that the nanoliposomes have good cytocompatibility.

### 3.4 Ultrasound mediated thrombolysis of pulmonary embolism *in vivo*

**FIGURE 4:**
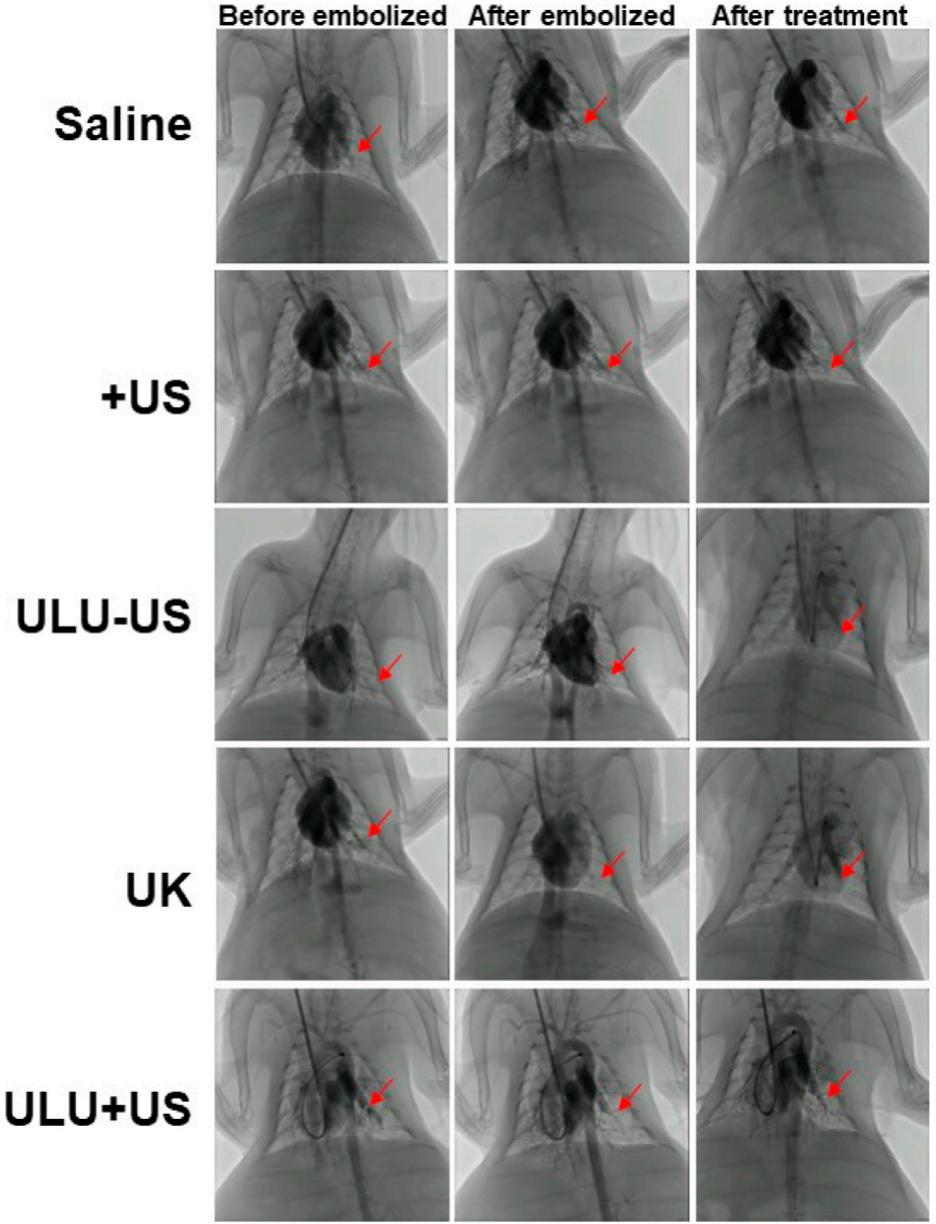
Digital subtraction imaging was used to evaluate the effect of pulmonary thrombolysis in rabbits receiving different treatment methods (Saline, US, ULU-US, United Kingdom, ULU + US, among the three groups (ULU-US, United Kingdom, ULU + US) had the same urokinase dose of 2,000 units, red arrows indicate recovery before and after pulmonary embolization and after treatment, *n* = 3).

Rabbits successfully constructed by jugular catheter were treated according to the established treatment regimen. It is can be seen that after a pulmonary embolism, the rabbits were given normal saline and blank liposome capsules without urokinase encapsulation (Figure 4 and Supplementary Figure S3–S7). After the treatment, the digital subtraction image of the lungs showed that the pulmonary embolism still existed, the blood vessels did not recover the blood flow, and the vital signs of the rabbits were gradually weakened and eventually died. However, when urokinase, a clinically used thrombolytic drug, was injected into rabbits with the same dose as urokinase nanoliposomes through the tail vein, and under the stimulation of ultrasound, there was no sign of blood flow recovery in pulmonary embolism within 30 min, and a little blood flow recovery in the lungs after 60 min, with no obvious therapeutic effect. It is worth noting that when the embolized rabbits received the ultrasound-responsive nanoliposome capsules, the blood flow of most of the blood vessels after artificial embolization was restored in the lungs 15 min later, and the blood flow of the originally blocked blood vessels was completely restored 30 min later, and the vital signs of the rabbits returned to the normal level. It is worth noting that we took blood from rabbits receiving different treatments and tested the concentration of fibrinogen in the blood (SI, Supplementary Figure S8). The results showed that when the embolized rabbits received a normal saline placebo, pure ultrasound and pure material treatment, the fibrinogen concentration in the blood was almost unchanged. While there was a slight decrease in blood fibrinogen concentrations when treated with pure urokinase, in contrast, when rabbits received ULU nanoliposomes and ultrasound treatment, blood fibrinogen concentrations decreased significantly. The results showed that it was upon stimulation by ultrasound that urokinase was released from the liposome capsules, which activated thrombolysis, resulting in a dramatic drop in fibrinogen concentrations. The observation of long-term survival showed that the rabbits had no abnormalities within 60 days after treatment, and the blood routine and biochemical structure showed that the rabbits had completely recovered.

### 3.5 Biocompatibility of ULU nanoparticles *in vivo*

**FIGURE 5:**
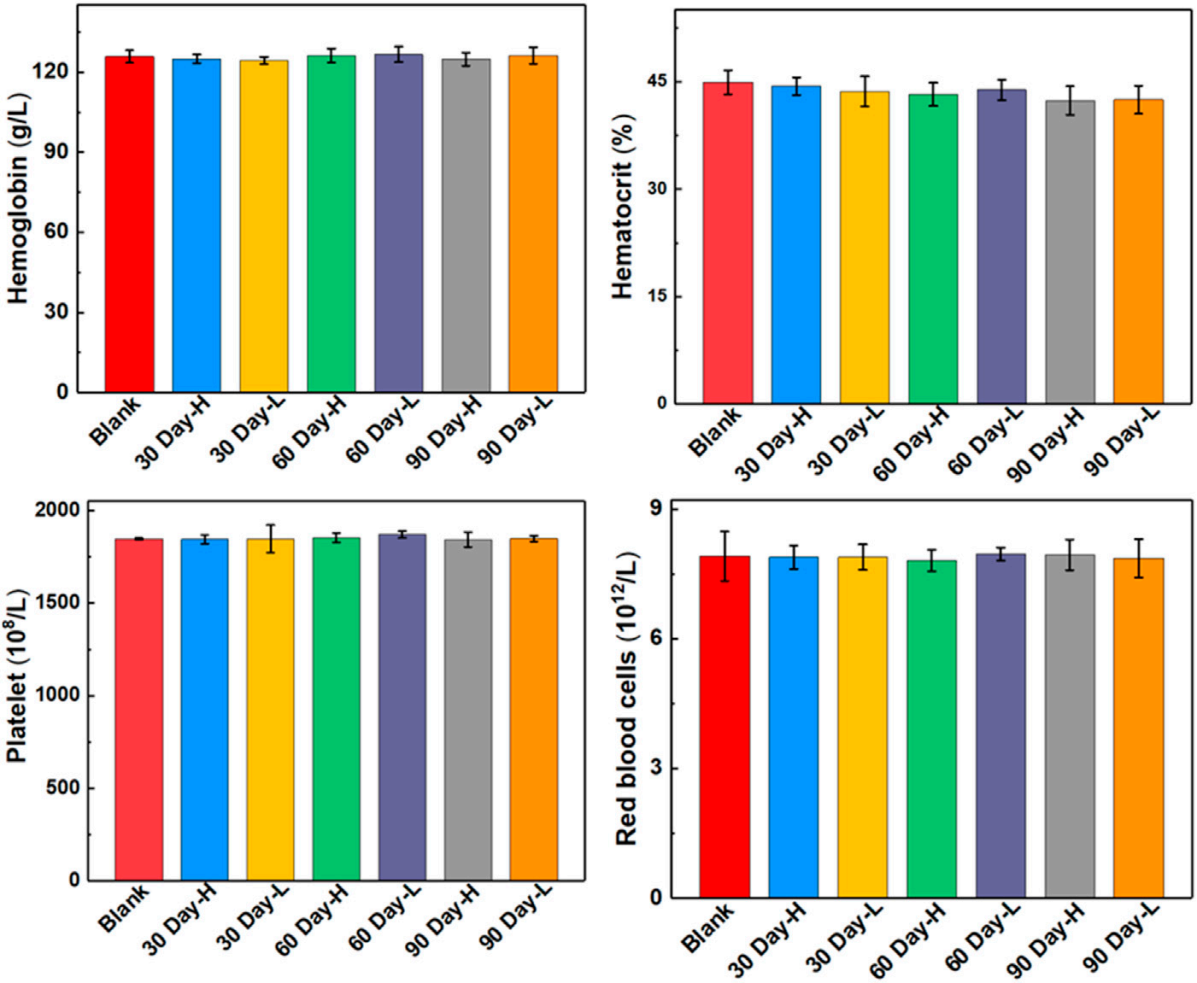
Blood routine indexes at different time points after ULU nanoliposome (20 mg/kg, *n* = 3) were injected into the tail vein of healthy mice.

**FIGURE 6:**
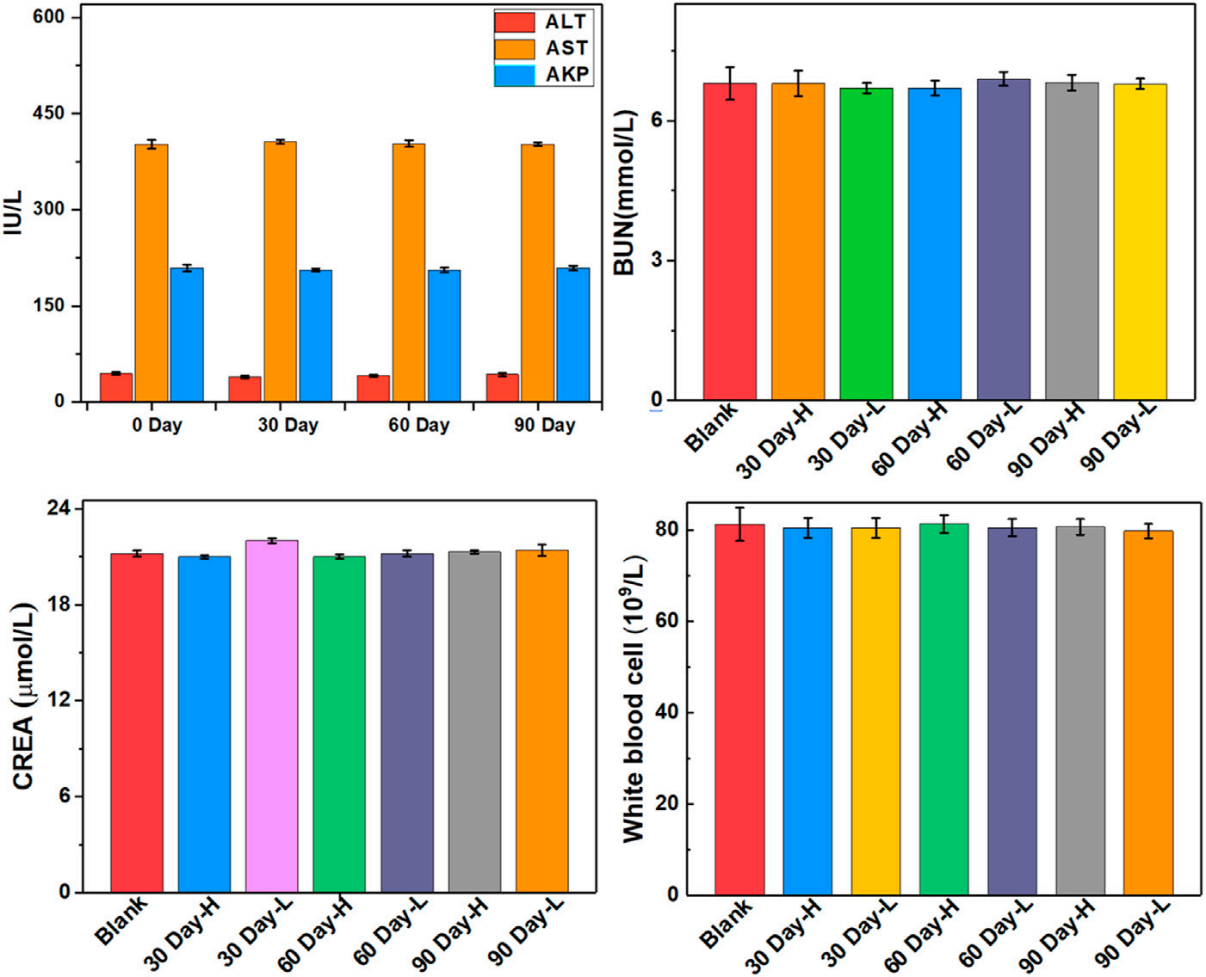
Important indicators of liver and kidney function at different time points after ULU nanoliposome (20 mg/kg, *n* = 3) were injected into the tail vein of healthy mice.

The biocompatibility and biotoxicity of the ULU nanoliposome were evaluated *in vivo*. No obvious behavioral abnormalities or weight loss were observed during the treatment of mice on 0 days and 90 days. Moreover, after treatment, H&E staining images showed that regions with necrosis or apoptosis were rarely detected in the murine major organs, including the heart, liver, spleen, and kidney in the different treatment groups (SI, Supplementary Figure S9). Additionally, analysis of long-term biotoxicity in mice revealed no significant differences in diversely vital blood parameters (Figure 5), and liver and kidney function indexes (Figure 6) among the 0 days, 30 days, 60 days, and 90 days. These results indicated the perfect biocompatibility of ULU nanoparticles for pulmonary embolism thrombolysis *in vivo*.

## 4 Conclusion

In conclusion, based on the fine chemical synthesis of molecular structure, nanoliposomes with the ultrasound-controlled release of urokinase were engineered and applied to the study of acute pulmonary embolism thrombolysis. The prepared ULU nanoliposomes have good colloid stability, blood compatibility, and cytocompatibility. More importantly, compared with the same dose of free urokinase, it has higher thrombolytic efficiency and safety, providing a good idea for the precise treatment of pulmonary embolism.

## Data Availability

The original contributions presented in the study are included in the article/Supplementary Material, further inquiries can be directed to the corresponding authors.
