# Supplementary material for: Construction of ultrasound-responsive urokinase precise controlled-release nanoliposome applied for thrombolysis
Source: Front Bioeng Biotechnol. 2022 Aug 9;10:923365. doi: 10.3389/fbioe.2022.923365 (PMC9396409; doi:10.3389/fbioe.2022.923365)
Supplement: Supplementary file 1 [file DataSheet1.docx]

**Construction of Ultrasound-Responsive Urokinase Precise Controlled-Release Nanoliposome Applied for Thrombolysis**

*Yongliang Fan^a,c^, Li Liu^b^, Fang Li^b^, Hang Zhou^b^, Yizhou Ye^c^, Chunping Yuan^d^, Hongli Shan^d^, Wangfu Zang^a^*, Yu Luo^d*^, Sijing Yan^e*^*

^a^ Department of Cardio-Thoracic Surgery, Shanghai Tenth People's Hospital, School of Clinical Medicine of Nanjing Medical University, Shanghai, 200072, P. R. China

^b^ Department of ultrasound medicine, Chongqing University Cancer Hospital, No. 181, Hanyu Road, Chongqing, P. R. China

^c^ Department of Cardiovascular Surgery, Shanghai General Hospital, Shanghai Jiao Tong University School of Medicine, No. 650 New Songjiang Road,Shang Hai, P. R. China

^d^ Shanghai Engineering Technology Research Center for Pharmaceutical Intelligent Equipment, Shanghai Frontiers Science Center for Druggability of Cardiovascular non-coding RNA, Institute for Frontier Medical Technology, Shanghai University of Engineering Science, Shanghai, P. R. China

^e^ Department of Ultrasound, Chongqing Hospital of Traditional Chinese Medicine, No. 6, Panxi seventh branch road, Jiangbei District, Chongqing, P. R. China

*** Correspondence:**

Wangfu Zang

zangwf@hotmail.com

Yu Luo

yuluo@sues.edu.cn

Sijing Yan

wennieyanyan@163.com

**Table S1：**The Zeta potential and hydrodynamic size of ULU nanoliposome dispersed in water.

| Sample | Zeta potential | Hydrodynamics size (nm) | PDI |
| --- | --- | --- | --- |
| ULU | -28.9 | 31.3±1.4 | 0.23 |

**Table S2:** The Encapsulation Efficiency (EE) and Loading Efficiency of urokinase in ULU nanoliposome.

| Sample | Encapsulation efficiency | Loading Efficiency |
| --- | --- | --- |
| ULU | 91.8% | 5.9% |


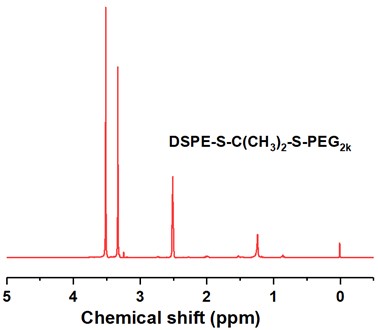


**Figure S1.** ^1^H NMR spectrum of DSPE-S-C(CH_3_)_2_-S-PEG_2k_.


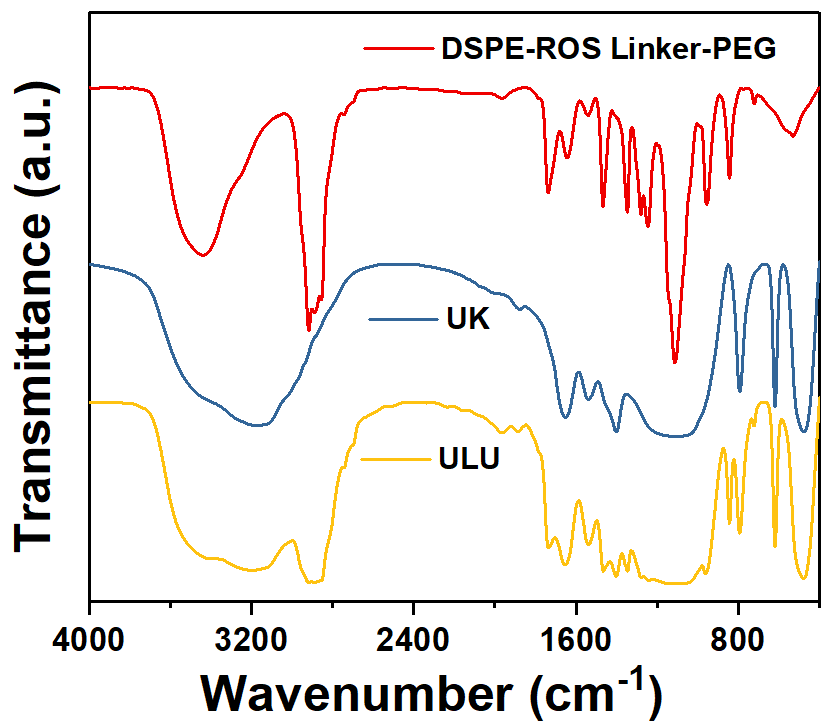


**Figure S2.** FTIR spectrum of the DSPE-ROS Linker-PEG, urokinase (UK), and urokinase encapsulated nanoliposome (ULU), respectively.


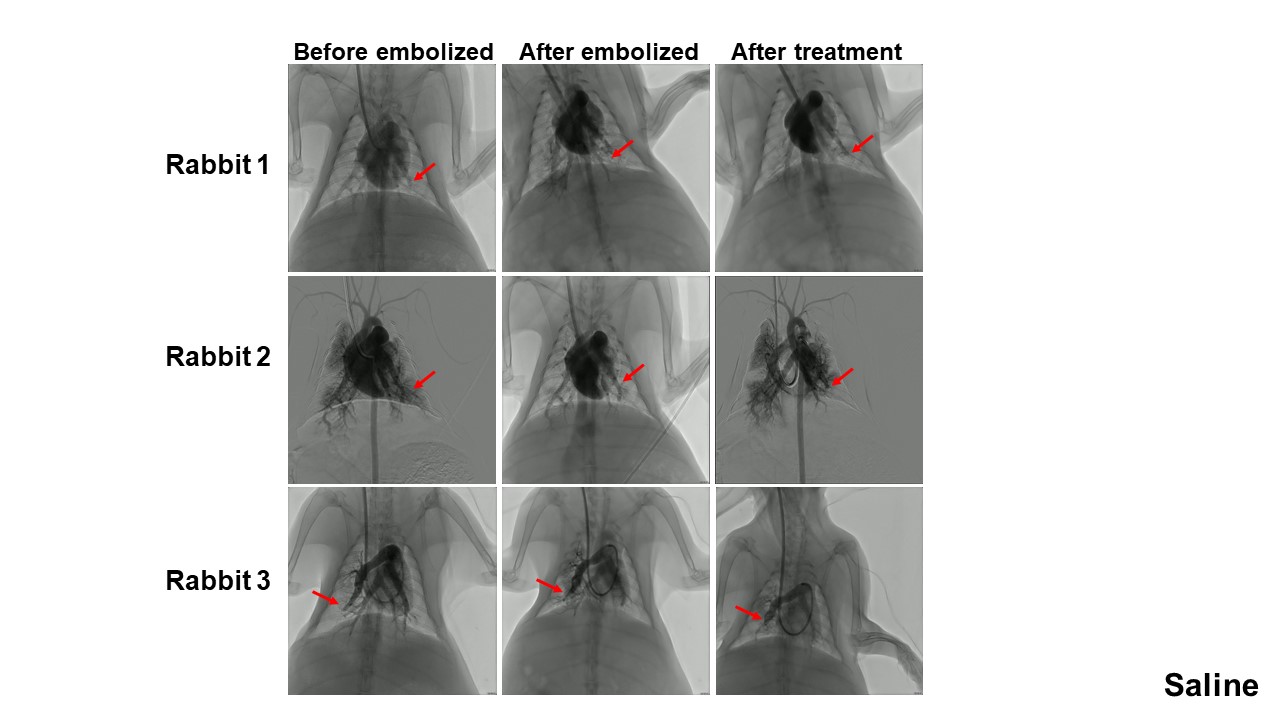


**Figure S3.** Digital subtraction imaging was used to evaluate the effect of pulmonary thrombolysis in rabbits receiving saline treatment (Saline: 2 mL, n = 3, red arrows indicate recovery before and after pulmonary embolization and after treatment).


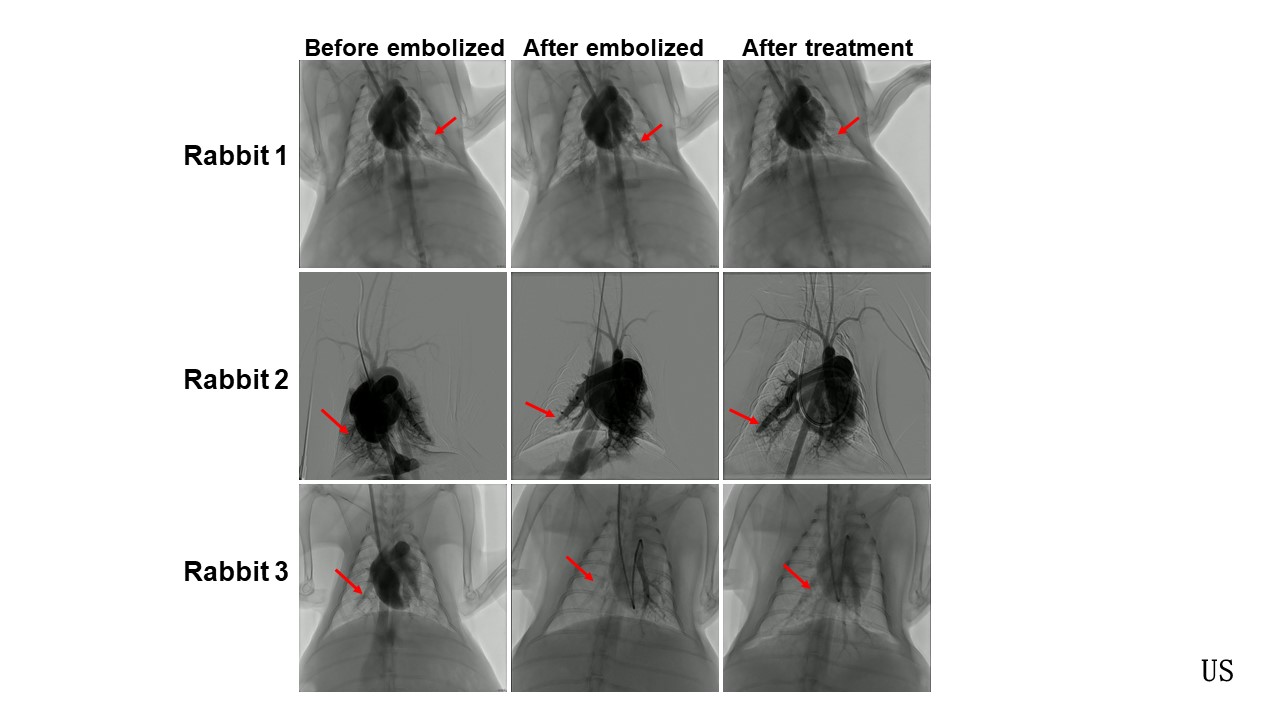


**Figure S4.** Digital subtraction imaging was used to evaluate the effect of pulmonary thrombolysis in rabbits receiving saline treatment (1.0 W/cm^2^, 5 min, n = 3, red arrows indicate recovery before and after pulmonary embolization and after treatment).


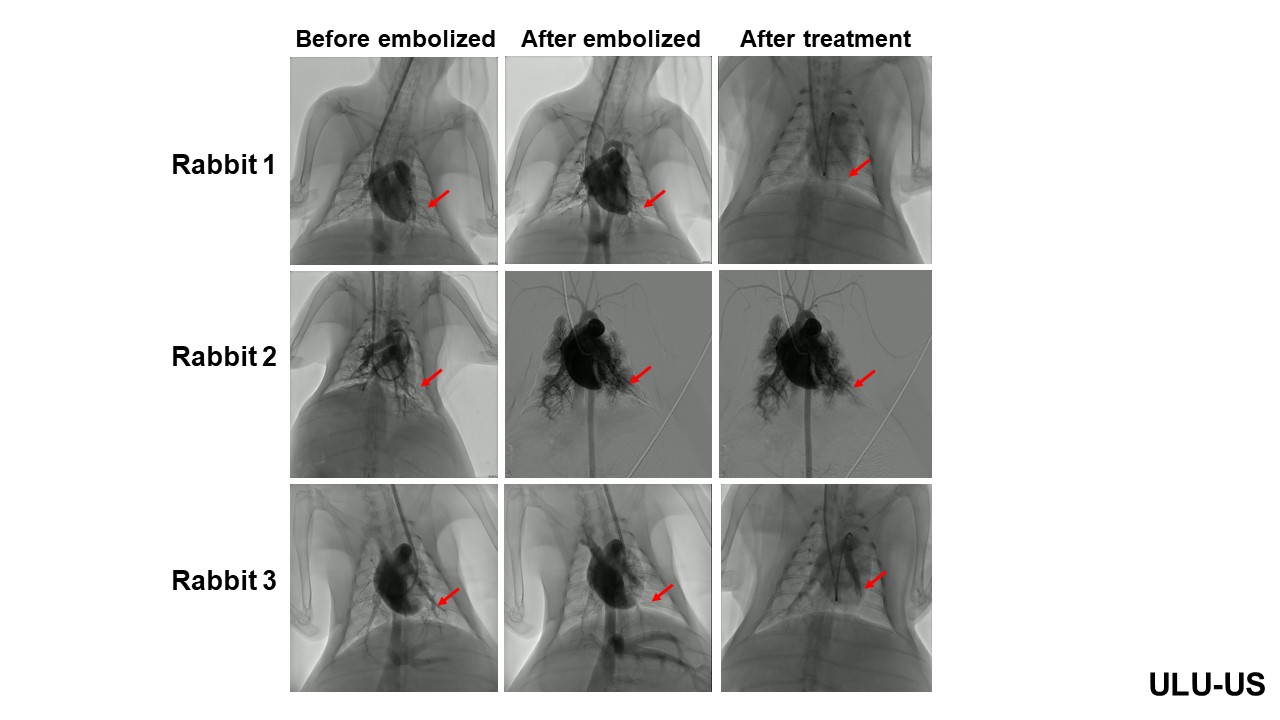


**Figure S5.** Digital subtraction imaging was used to evaluate the effect of pulmonary thrombolysis in rabbits receiving ULU without US treatment (with the urokinase dose of 2,000 units dispersed into 2 mL saline, red arrows indicate recovery before and after pulmonary embolization and after treatment).


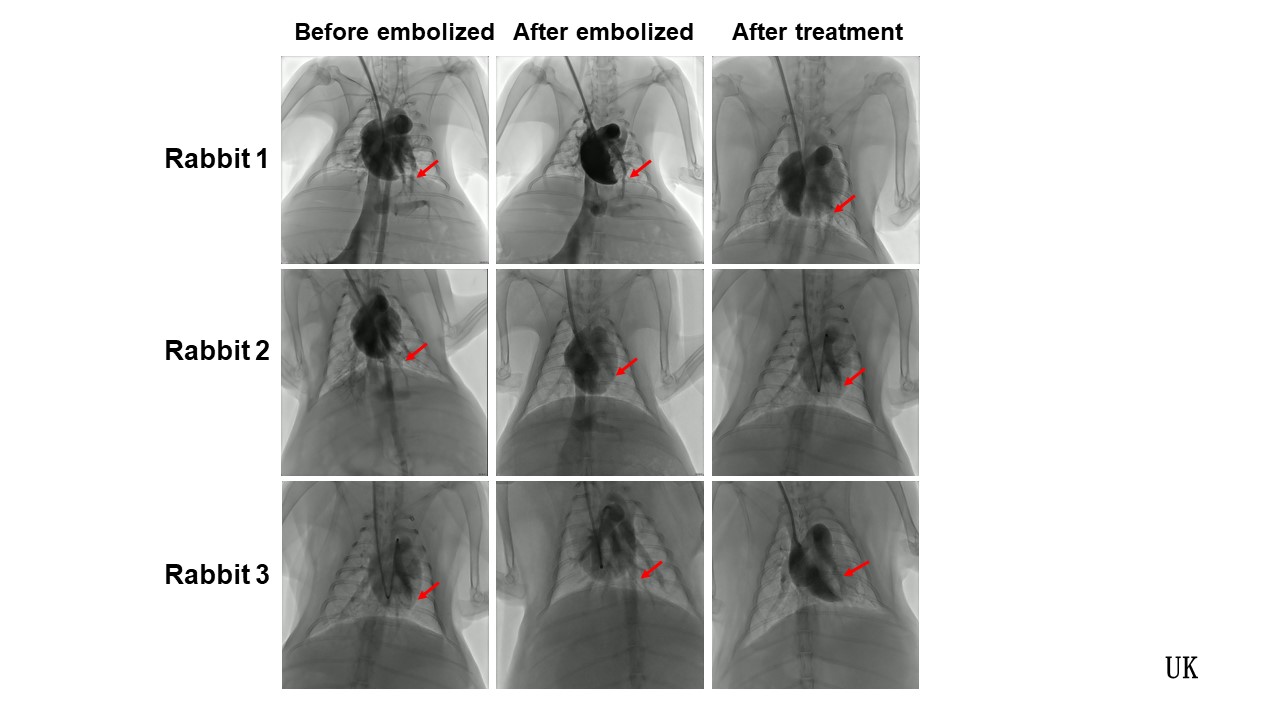


**Figure S6.** Digital subtraction imaging was used to evaluate the effect of pulmonary thrombolysis in rabbits receiving UK treatment (with the urokinase dose of 2,000 units dispersed into 2 mL saline, n = 3, red arrows indicate recovery before and after pulmonary embolization and after treatment).


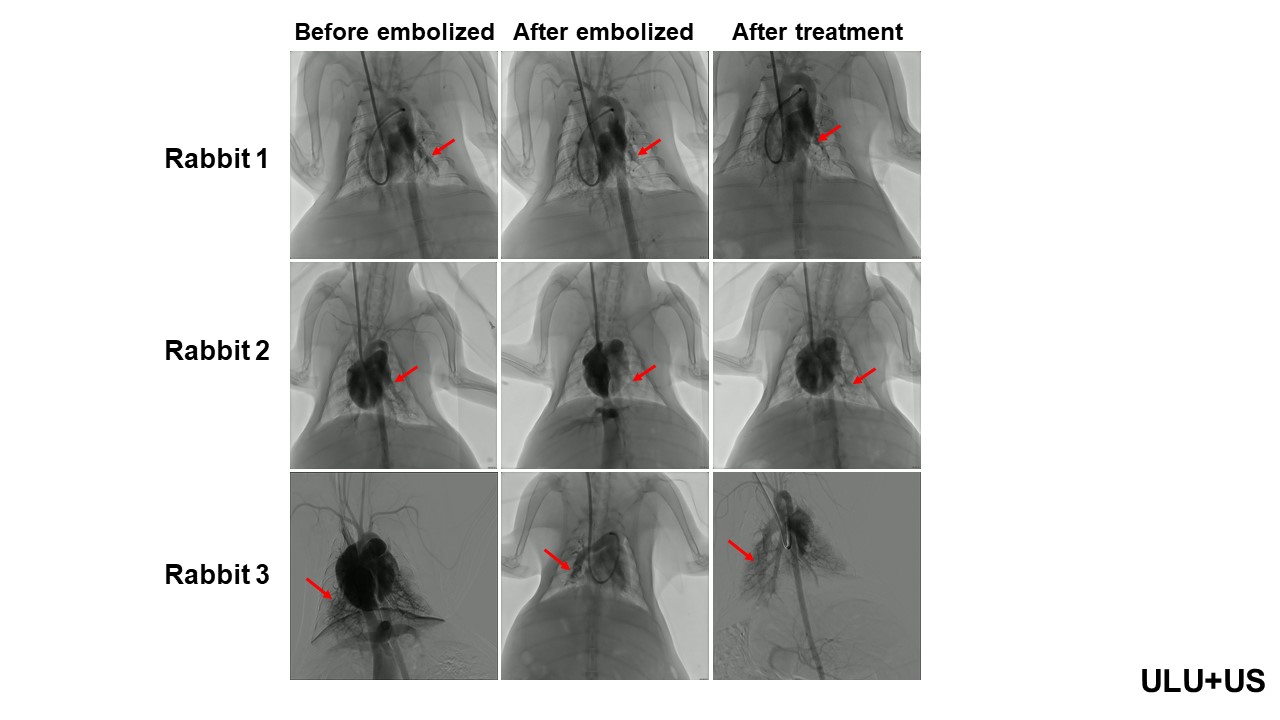


**Figure S7.** Digital subtraction imaging was used to evaluate the effect of pulmonary thrombolysis in rabbits receiving ULU with US treatment (1.0 W/cm^2^, 5 min, with the urokinase dose of 2,000 units dispersed into 2 mL saline, n = 3, red arrows indicate recovery before and after pulmonary embolization and after treatment).

**Figure S8.** Changes of fibrinogen concentration in blood of pulmonary embolism model rabbits after receiving different treatments (Saline, +US, ULU-US, UK, ULU+US, respectively).


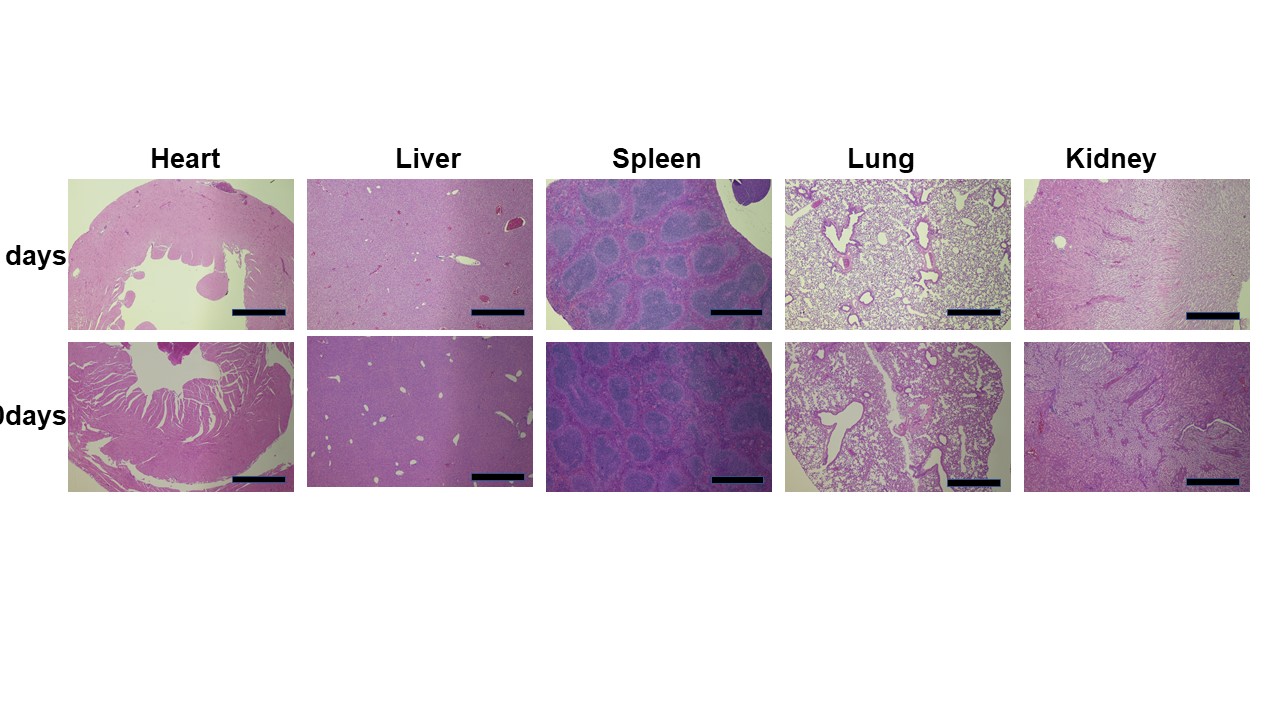


**Figure S9.** H&E staining images of Heart, Liver, Spleen, Lung, and Kidney from mice after different treatments for 0 days and 90 days, scale bar = 1000 μm.
